# Ventricular tachycardia isthmus identification in postinfarction case using abnormal activity and functional substrate mapping with reference to line of block

**DOI:** 10.1016/j.hrcr.2025.06.030

**Published:** 2025-07-03

**Authors:** Yusuke Sakamoto, Hiroyuki Osanai, Yuichiro Sakai, Yoshiki Sogo, Eiji Yoshida, Yoshihito Nakashima, Hiroshi Asano

**Affiliations:** Department of Cardiology, Tosei General Hospital, Seto, Aichi, Japan

**Keywords:** Isochronal late activation mapping, Line of block, Rotational activation pattern, Ventricular abnormal activities, Ventricular tachycardia

**Key Teaching Points**

• A line of block (LOB), visualized by adjusting “Early Meets Late” thresholds during sinus rhythm, can delineate the boundaries of critical ventricular tachycardia (VT) circuits, even in the absence of mapping during tachycardia.
• The convergence of ventricular abnormal activities, isochronal late activation mapping (ILAM), isochronal crowding, and rotational activation pattern (RAP) at the end of the LOB, particularly when RAP overlaps with clustered abnormal activities, suggests a critical isthmus and enables more precise targeting for ablation.
• Combining LOB, ventricular abnormal activities, ILAM, RAP, and pace mapping provides a practical and reliable strategy for substrate-guided VT ablation in patients with unmappable VT.
• Targeting sites where multiple substrate markers spatially overlap allows for efficient isthmus localization, reduces the need for extensive pace mapping, and leads to safe and short ablation times.

## Introduction

In recent years, substrate assessment using 3-dimensional (3D) mapping has become increasingly important as an ablation strategy for ventricular tachycardia (VT). In particular, for cases with unstable hemodynamics during VT, substrate mapping during sinus rhythm is useful in estimating the critical isthmus, which is key to effective treatment.

Ventricular abnormal activity detection (VAAD) maps are characterized by their ability to visualize lines of block (LOB) and comprehensively detect local abnormal ventricular activities, thereby contributing to ascertaining the structural and electrical properties of the VT substrate.^1^

Furthermore, isochronal late activation mapping (ILAM), a type of functional substrate mapping, is considered useful in estimating slow conduction zones in VT circuits by visualizing regions of delayed conduction as isochronal crowding (IC).^2^

In addition, rotational activation patterns (RAPs) indicate sites where patterns of excitation propagation curve inward at angles of ≥90°, immediately above or on the margin of IC, and a correlation with the critical isthmus has been reported. Retrospective analyses have shown that RAP is observed in approximately 70% of VT critical isthmuses, most of which correspond to the exit.^3^

In this case, analysis of post–posterior infarction VT using a combination of LOB-focused VAAD mapping and functional substrate mapping (ILAM and RAP) allowed for the identification of the optimal ablation site, and VT was successfully treated. We present the following case, which suggests that a multifaceted substrate assessment is beneficial.

## Case report

An 80-year-old man with a history of posterior myocardial infarction 11 years earlier had been monitored for chronic ischemic cardiomyopathy. Four years ago, a subcutaneous implantable cardioverter-defibrillator was implanted at another hospital after the patient presented with VT. However, the use of amiodarone was problematic owing to liver disorder, and the patient had recently experienced multiple subcutaneous implantable cardioverter-defibrillator shocks for frequent episodes of VT, each causing immediate hemodynamic compromise. Therefore, the patient was referred to our hospital for catheter ablation.

An echocardiogram revealed a left ventricular ejection fraction of 31%, left ventricular enlargement, and reduced contractile performance with advanced left ventricular dilatation measuring 66 mm in diameter. Mapping during VT was deemed problematic owing to hemodynamic instability; therefore, we attempted to estimate the critical isthmus using substrate mapping.

Ablation was initiated using a transseptal approach. CARTO 3 version 8 (Biosense Webster, Diamond Bar, CA) was used for navigation, and OPTRELL was used for mapping.

Upon creating an initial voltage map (voltage range 0.1–0.5 mV), an extensive low-voltage zone (LVZ) was noted from the posterior wall to the apex.

**Figure 1:**
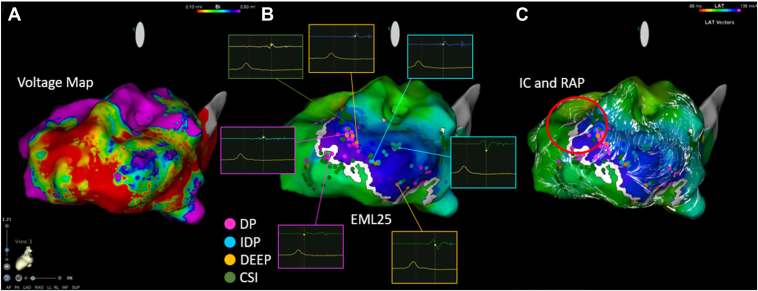
VAAD map and functional substrate map–driven visualization of the VT isthmus. **A:** On a voltage map during sinus rhythm, an extensive low-voltage zone (LVZ) is noted stretching from the posterior wall to the apex. **B:** By gradually adjusting the lower threshold using the Early Meets Late (EML) function, a clear and fixed line of block (LOB) across the LVZ center is visualized. DP, IDP, and DEEP are manually tagged for added automatic detection using the CSI function. **C:** IC and RAP are noted at the end of the LOB on isochronal late activation mapping (ILAM), and findings are obtained that are strongly suggestive of a critical isthmus. CSI = complex signal identification; DEEP = decrement-evoked potential; DP = delayed potential; IDP = isolated delayed potential; IC = isochronal crowding; RAP = rotational activation pattern; VAAD = ventricular abnormal activity detection.

Next, we prioritized visualizing the LOB to construct a VAAD map. By gradually adjusting the lower threshold using CARTO’s Early Meets Late (EML) function, a clear, linear EML image was visualized across the left ventricular cavity (Figure 1B). The optimal threshold visibility was achieved at 25%, and this setting was maintained. The LOB was determined to be of the linear type, consistent with the report by Nishimura et al.^4^

After LOB visualization, delayed potentials (DPs), isolated DPs, and decrement-evoked potentials (DEEPs) were manually tagged based on signals acquired with the OPTRELL catheter. DEEPs, in particular, are late potentials appearing owing to S1–S2 stimulation, and Jackson et al^5^ have reported that they correlate highly with the diastolic pathway in the VT circuit. In addition, we used the CARTO 3 V8 complex signal identification function for automatic extraction of abnormal activities based on potential width and polarity reversal. Although multiple abnormal activity clusters were identified on the VAAD map, no single critical isthmus was clearly defined, prompting us to proceed with functional substrate mapping.

ILAM revealed IC at the distal end of the LOB, suggesting a reduced conduction rate in that area. Visualization of local conduction using CARTO 3 V8’s local activation time velocity vectors showed an area where patterns of excitation propagation were seen that curved inward at angles of ≥90° at the distal end of the LOB and immediately above the IC. This pattern was judged to represent an RAP (Figure 1C).

**Figure 2:**
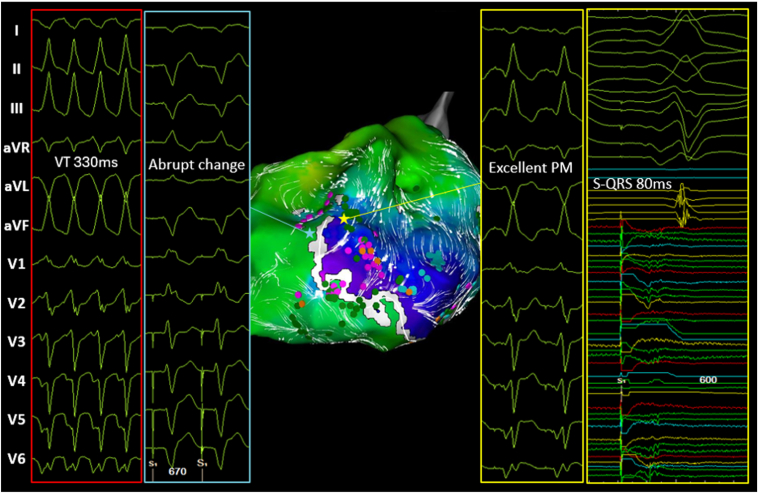
Confirmation of the VT exit by precise pace mapping. On pace mapping, an excellent pace map match of 80 ms for the S-QRS interval is observed at the end of the LOB, and an abrupt change appears in the neighboring site. This is determined to be the critical isthmus near the exit. LOB = line of block; PM = pace map; VT = ventricular tachycardia.

Pace mapping performed at this site indicated an S-QRS interval of 80 ms, which was an excellent match with the clinical VT. Based on the finding of abrupt changes in pace mapping at the neighboring site (opposite LOB), we concluded that this site represented the critical isthmus near the exit (Figure 2).

**Figure 3:**
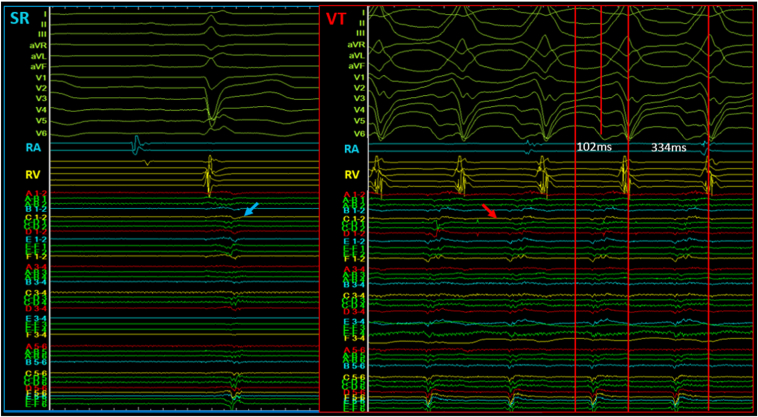
VT induction and elimination by targeted ablation. When inducing a VT by placing a multielectrode catheter at the site corresponding to the pace map, DP (*blue arrow*) changes to presystolic potential (*red arrow*), which fills most of the diastolic phase, and we conclude that this is the VT critical isthmus. DP = delayed potential; RA = right atrium; RV = right ventricle; SR = sinus rhythm; VT = ventricular tachycardia.

**Figure 4:**
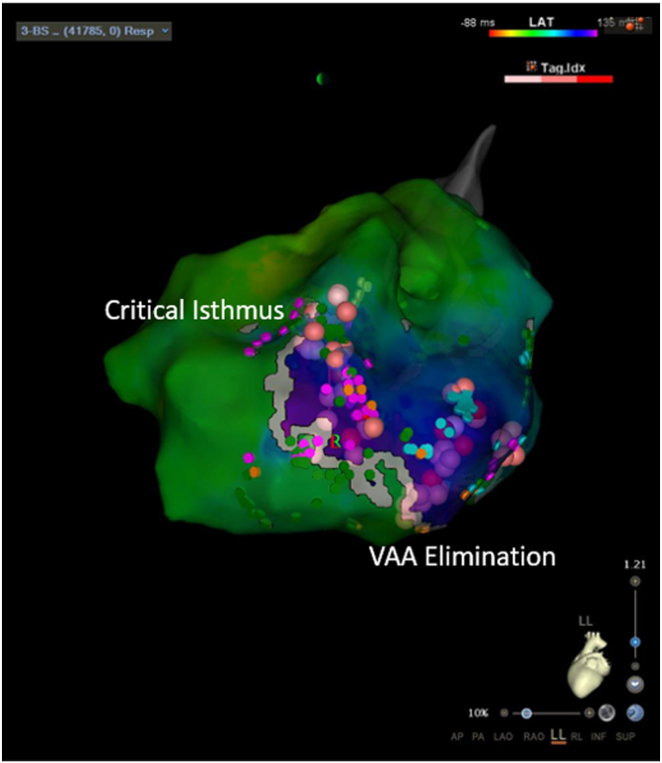
Ablation sites targeting the ventricular tachycardia (VT) critical isthmus, identified via multimodality substrate mapping. Ablations were delivered at the site identified by the combination of VAAD mapping and functional substrate mapping. This region also exhibited an excellent pace map match (S-QRS = 80 ms). Mid-diastolic potentials were recorded during induced VT. After ablation at this site, VT was rendered completely noninducible. An additional ablation was performed to further eliminate abnormal activity. On postablation mapping, the previously observed RAP had disappeared, and no new RAP was identified, thereby supporting successful substrate modification. RAP = rotational activation pattern; VAA = ventricular abnormal activity; VAAD = ventricular abnormal activity detection.

When inducing a VT by placing a multielectrode catheter on this site, DP (blue arrow) changed to a presystolic potential (red arrow), which fills most of the diastole phase, and we concluded that this was the VT critical isthmus (Figure 3). Owing to hemodynamic failure, we terminated VT by pacing; however, after the ablation of this site, VT was no longer inducible once the critical isthmus was removed. When abnormal electrograms were identified via VAAD mapping, additional ablations were performed to minimize the risk of recurrence (Figure 4). Postablation mapping confirmed the disappearance of preexisting RAP and the absence of new RAP. No recurrent arrhythmia was seen during a period of more than 1 year since the ablation, and the patient has been stable.

## Discussion

In this case, we estimated the critical isthmus through multifaceted substrate assessment using a combination of VAAD mapping and functional substrate mapping for unmappable VT and achieved noninducibility of VT through targeted ablation. Furthermore, it is important that this case showed crowding of ventricular abnormal activities near the LOB and that an RAP overlapped with the same site on the functional map. In contrast, although abnormal activities (local abnormal ventricular activities, late potentials, or DEEP, etc) may be distributed along a critical isthmus, they also often appear atypically scattered around scar tissue, and there are limits to their selective targeting. In addition, because IC may also extend over multiple sites or appear as a band-like pattern on ILAM, there are limitations in that it is difficult to determine a treatment target.^2^^,^^3^ In contrast, RAP is a localized phenomenon appearing in the center of the IC and is believed to be of high specificity. In fact, the overlap of abnormal activities and RAP on the same site substantially increases its diagnostic reliability for the critical isthmus, and the ablation target can be clarified. Accordingly, a multimodality strategy integrating comprehensive detection of abnormal activities (VAAD mapping) and functional assessment (ILAM and RAP) is an important future key to substrate-based VT ablation.

For this case, we first identified, using voltage mapping, an extensive LVZ that stretched from the posterior wall to the apex. By gradually adjusting the lower threshold of the EML function, we visualized a fixed LOB across the LVZ. The formation of a functional or anatomic block line is essential to maintaining VT, and it is highly significant that a construction that corresponds to that can be identified during sinus rhythm. Such an LOB has been regarded as important in recent years as a boundary restraining circuit structures in 3D, and Nishimura et al^4^ reported that 74% of VT isthmuses were bounded by an LOB during baseline rhythm.

Next, we used VAAD mapping to visualize abnormal activities (DP, isolated DP, and DEEP) distributed along the LOB. It is important to note that, in this case, ventricular abnormal activities concentrated near the LOB also support the presence of a critical isthmus.

On ILAM, which was created as a functional map, IC was seen at the end of the LOB, and RAP was also mapped onto the same site. RAP is a structure formed by reentry into the tissue where the conductivity rate is relatively sustained, and Komatsu et al^3^ reported that RAP is present in 70% of VT circuits and corresponds to the exit in 71% of these.

Research by Tung et al^6^ showed that most reentrant VT circuits have 3D structures, and it is also evident that some clinical circuits exhibit simultaneous excitation and transmural propagation in both the inner and outer membranes. This suggests that 2-dimensional mapping alone may be insufficient.

However, in 3DVT, both the depth and lateral boundary may be specified by the LOB, and the reentry site may be in the nearby region. It has been reported that 79% of 3DVT depth boundaries are consistent with the LOB,^4^ and based on the confirmation of RAP in this case, it is believed that the main part of the VT circuit exists on the endocardium side.

Furthermore, we recorded the mid-diastolic potential of 100 ms prior to the QRS onset when inducing VT, which is strongly suggestive of reentry being the VT mechanism. Likely, the site where multiple substrate indices (LOB, abnormal activities, ILAM, RAP, mid-diastolic potential) converge is a critical isthmus. By having confirmed an excellent match of S-QRS interval (80 ms) and an abrupt change in neighboring sites on pace mapping, it was diagnosed as an exit-neighboring isthmus, and triggering could be halted through ablation.

In addition, CARTO V8 complex signal identification and local vector visualization were instrumental in detecting abnormal activities and RAP in this case and proved useful in identifying the critical isthmus.

The key point is that, although pace mapping had a critical role in confirming the VT isthmus, the identification of RAP at the distal end of the LOB, overlapping with IC, allowed us to target a highly specific area. This RAP-guided strategy enabled us to minimize the number of pace mapping attempts and optimize procedural efficiency, particularly in the setting of hemodynamic instability. The sequential narrowing of the ablation target using LOB, VAAD mapping, ILAM, and finally RAP provided a structured framework and led to a successful and safe isthmus identification.

In summary, this case highlights that centering the mapping approach around the LOB enables a structured and time-efficient process for identifying the VT critical isthmus. By integrating VAAD mapping and functional substrate mapping, including RAP analysis, we were able to streamline the workflow and reduce the overall procedure time without complications. Automated features of CARTO V8, such as abnormal activity detection and local excitation vector visualization, proved particularly effective.

## Conclusion

In this case, we identified the critical isthmus through multifaceted assessment using VAAD mapping and functional substrate mapping for unmappable VT, and we were able to terminate VT through accurate ablation. LOB-focused analysis is essential, in particular, for understanding the VT circuit and the essence of the treatment, and this case provides clinical evidence supporting its practical value.

## Disclosures

The authors have no conflicts of interest to disclose.
